# Rare Encounter: Giant Hemangiopericytoma of Thigh in a Young Male

**DOI:** 10.7759/cureus.59514

**Published:** 2024-05-02

**Authors:** Abhilasha Bhargava, Bhushan Jajoo, Tejshri Telkhade, Aditya Patel, Jayashree Bhawani

**Affiliations:** 1 General Surgery, Jawaharlal Nehru Medical College, Datta Meghe Institute of Higher Education and Research, Wardha, IND; 2 Surgical Oncology, Jawaharlal Nehru Medical College, Datta Meghe Institute of Higher Education and Research, Wardha, IND; 3 Radiation Oncology, Jawaharlal Nehru Medical College, Datta Meghe Institute of Higher Education and Research, Wardha, IND; 4 Pathology, Jawaharlal Nehru Medical College, Datta Meghe Institute of Higher Education and Research, Wardha, IND

**Keywords:** pericytes, thigh swelling, myofibromatosis, thigh, soft tissue tumor, hemangiopericytoma

## Abstract

A rare tumor called hemangiopericytoma develops from the pericytes, the cells that surround blood vessels. They frequently grow slowly and might be asymptomatic initially. Although they can develop anywhere in the body, these tumors are most frequently found in the head, pelvis, and legs. This uncommon tumor originates in soft tissues like fat, muscles, tendons, nerves, blood vessels, and other fibrous tissues. The tumor in adolescence can be benign or malignant; it frequently develops in the bones but has the potential to metastasize to the lungs. Imaging tests, such as MRIs or CT scans, are commonly used in diagnosis to determine the location and size of the tumor. We present a case of a 23-year-old male who complained of swelling in his left thigh that had persisted for two years. He underwent multiple biopsies which were inconclusive until wide local excision of the swelling was done. On histopathology, the excised tumor was suggestive of hemangiopericytoma. The patient was advised of radiotherapy for completion of the treatment.

## Introduction

Stout and Murray first identified and named the unusual mesenchymal neoplasm known as hemangiopericytoma in 1942, but it was not until 1949 when Stout reported 25 further cases that the tumor gained broad notice [1]. A tumor can develop at any place with proper blood supply. Pericytes, contractile spindle cells that surround capillaries and post-capillary venules, are thought to be the source of hemangiopericytoma. The tumor often consists of uniformly elongated cells encircling a dense, branching network of capillaries with thin walls that come in a variety of sizes and forms [2,3]. However, because various soft tissue neoplasms may show regions of abundant “hemangiopericytoma-like” vascularity, it can be challenging to make the histological diagnosis of hemangiopericytoma [4]. Hemangiopericytomas in the thoracic cavity, head and neck region, genitourinary tract, peritoneal cavity, retroperitoneum, soft tissues of the trunk and extremities, and the thoracic spine have been reported in publications [1]. We are presenting a case of hemangiopericytoma of the thigh in a young male patient with presentation as swelling and no other associated symptoms. Hemangiopericytoma is typically treated with surgical tumor excision followed by adjuvant radiotherapy. However, because of their location and propensity to return, these tumors can be difficult to completely remove, so in certain cases, additional treatments like radiation therapy or targeted therapy may be advised. Regular follow-ups are essential to monitor for any signs of recurrence or metastasis.

## Case presentation

**Figure 1:**
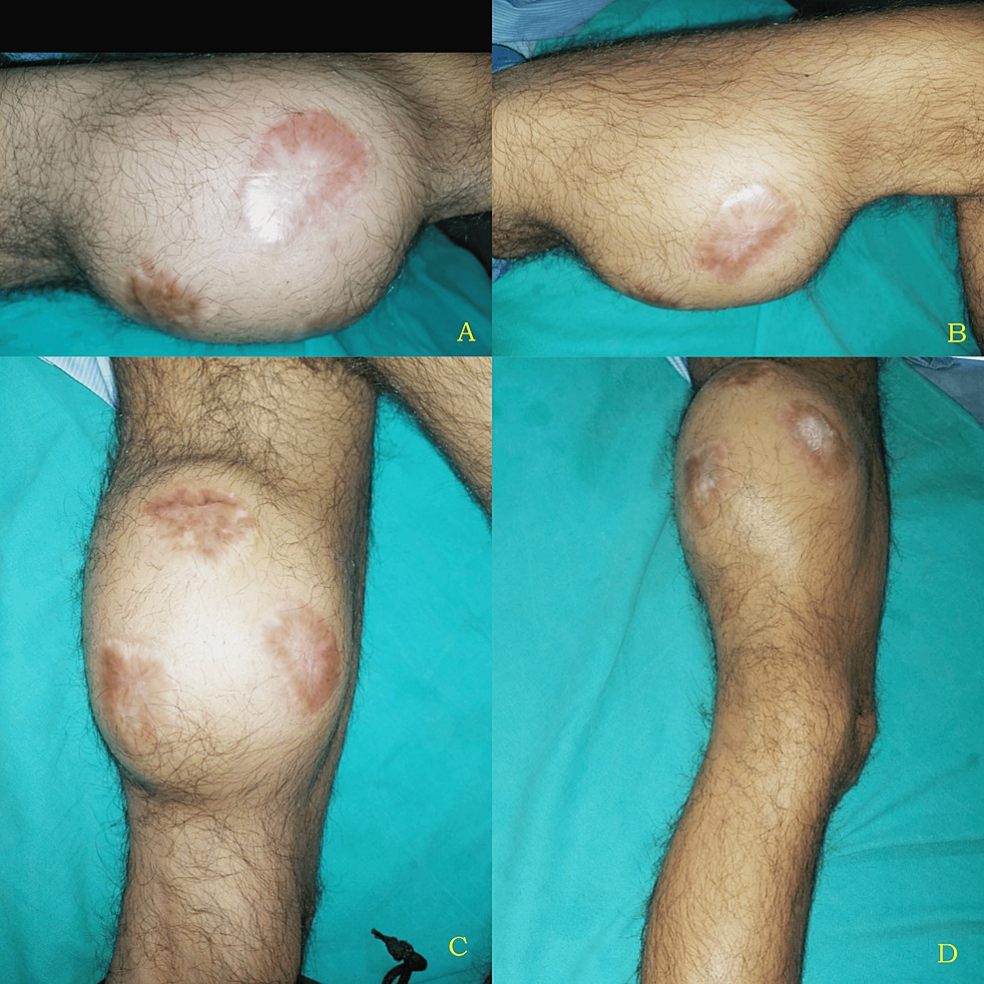
(A-D) Clinical image showing swelling over the thigh

A 23-year-old male presented with chief complaints of swelling in his left thigh for two years. It was insidious in onset and gradually progressive and found to be associated with difficulty in walking. The patient was non-alcoholic and non-smoker. The patient reported the swelling of the thigh not to be associated with pain, tenderness, or any history of trauma (Figures 1A-1D).

**Figure 2:**
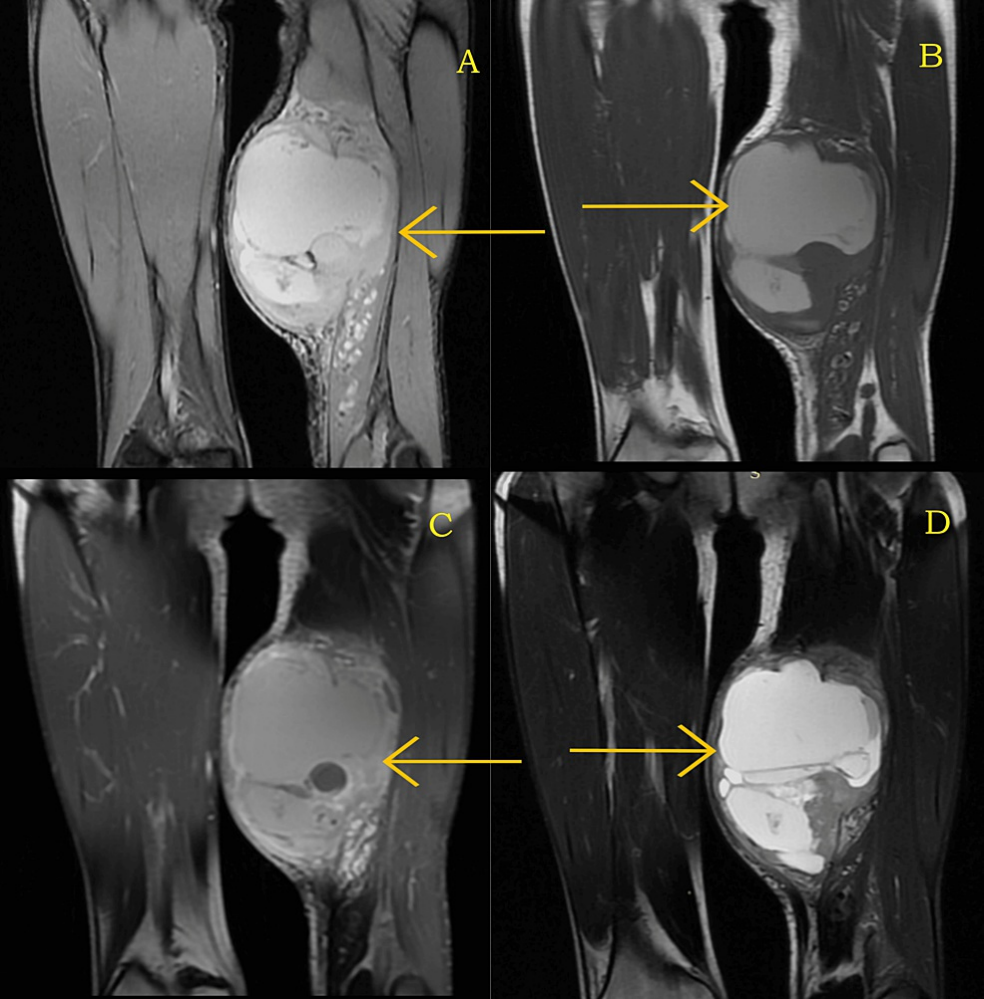
(A-D) Magnetic resonance images of the tumor in the left thigh

The patient did not have a positive history of any major illness. The patient was subjected to a biopsy of the lesion, which was suggestive of a possibility of synovial sarcoma, tumor cells were reported positive for S100, SS-18, Pan CK and reported negative for SOX-10, SMA, CD34, ERG, HMB 45, CD117, Melan A and STAT 6. The ki67 index was 25%. Immunohistochemical markers, erythroblast transformation specific-related gene (ERG), and CD34 highlighted vasculature which was indicative of hemangioma. The patient underwent magnetic resonance imaging (MRI) of the left thigh from a local hospital which was suggestive of a relatively well-defined heterogeneously enhancing solid cystic lesion of CC 15.1 × AP 9.7 × TR 11.5 centimeters predominantly cystic mass lesion observed involving adductor magnus of left mid-thigh without obvious bony destruction with dilated tortuous intramuscular vessels in semi tendinosis distal to the lesion (Figures 2A-2D).

**Figure 3:**
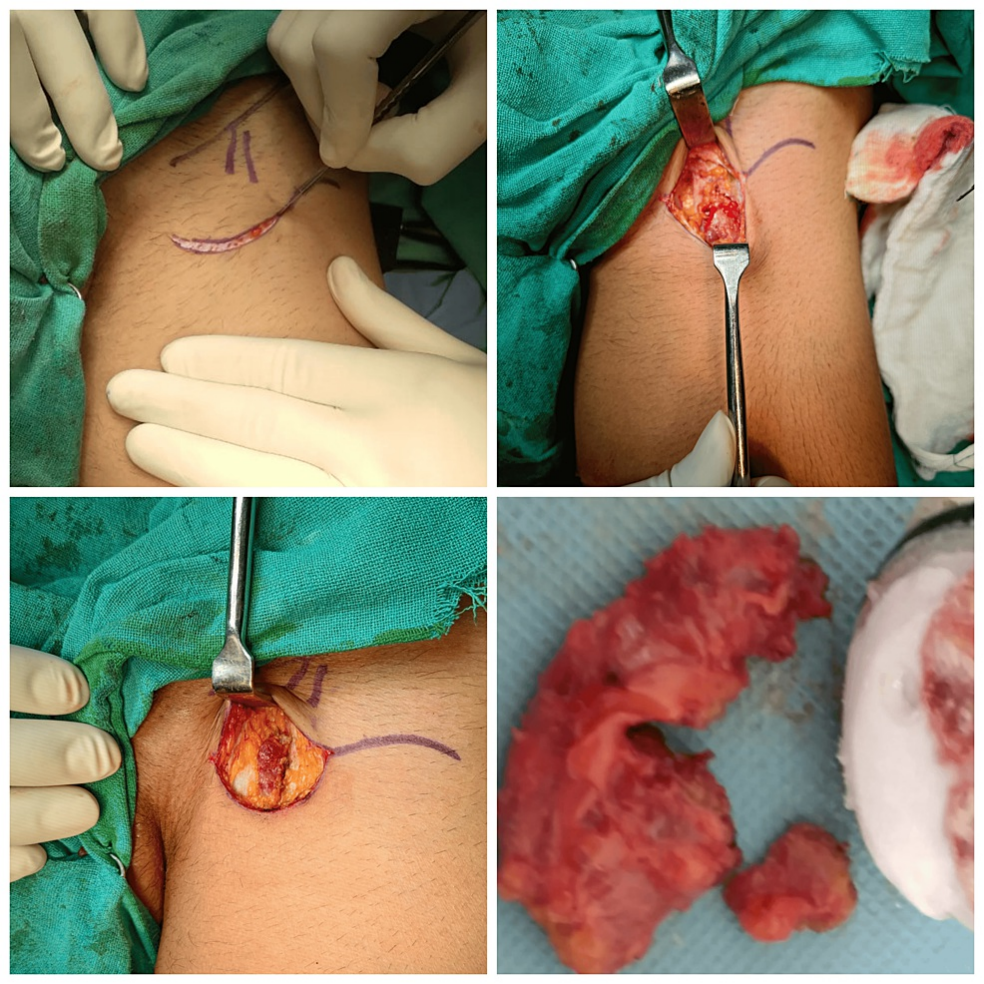
Intraoperative images of Ipsilateral superficial inguinal lymph node dissection

**Figure 4:**
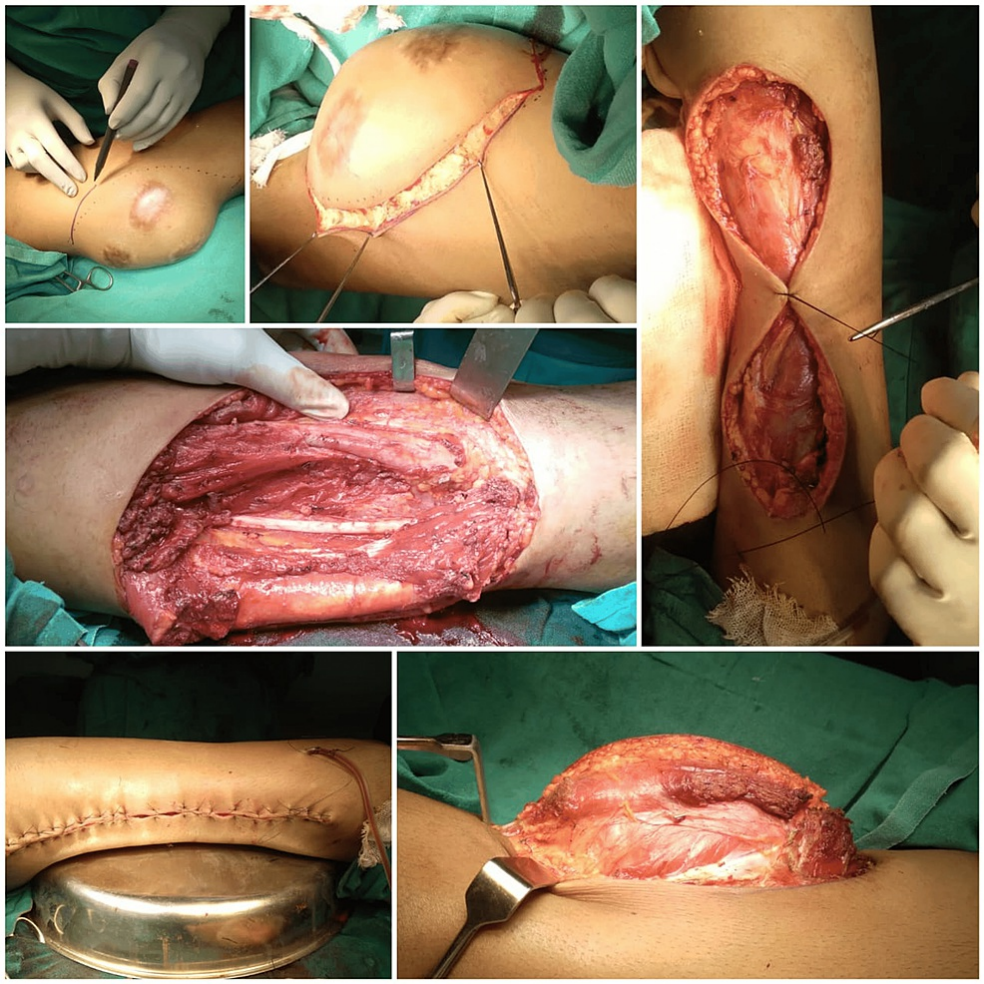
Intraoperative photos of wide local excision of the tumor

**Figure 5:**
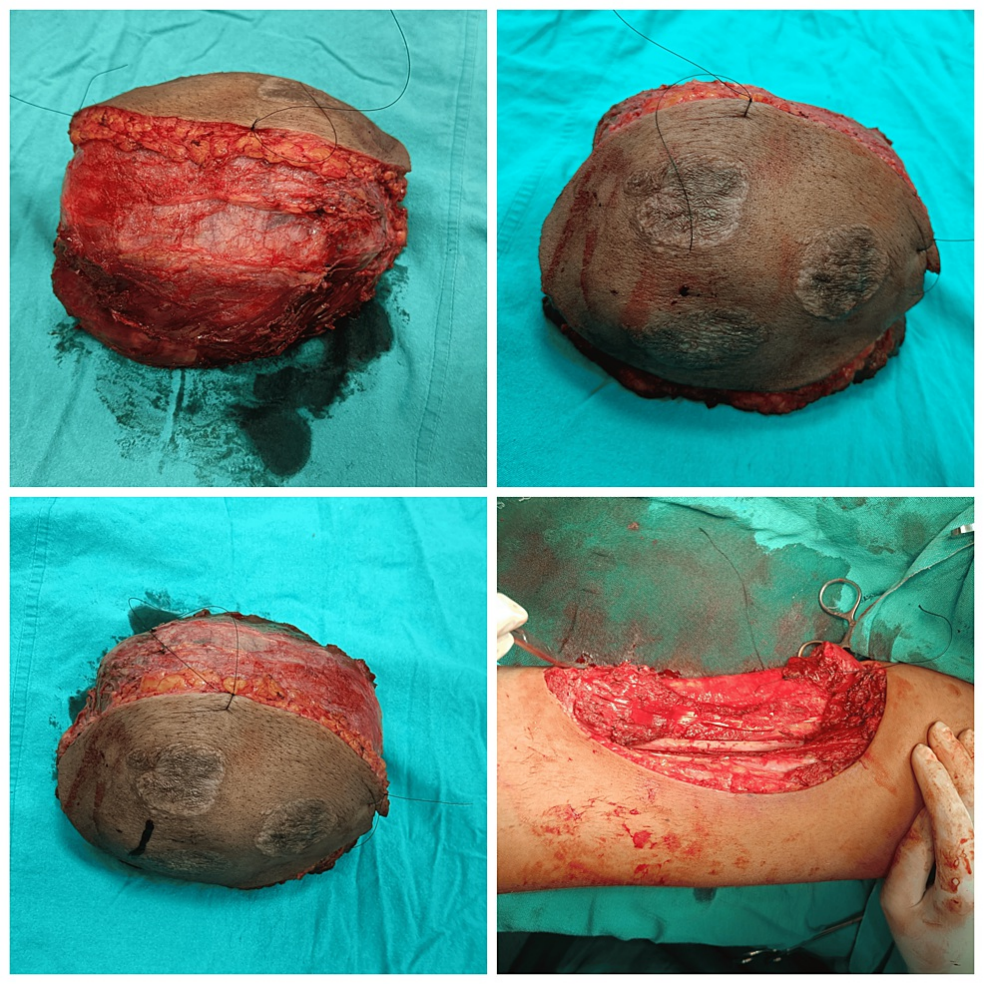
Excised specimen of hemangiopericytoma of the thigh

No evidence of involvement of neurovascular bundles was found. These features were suggestive of neoplastic etiology with internal hemorrhage such as soft tissue sarcoma. A computed tomography (CT) scan of the thorax was negative for lung metastasis. An attempt at Trucut biopsy was performed which was inconclusive. The patient was further planned for wide local excision and regional nodal sampling. The patient underwent wide local excision of the tumor along with ipsilateral inguinal lymph node dissection. The tumor was found to be intramuscular over the medial aspect of the left thigh (Figures 3-5).

**Figure 6:**
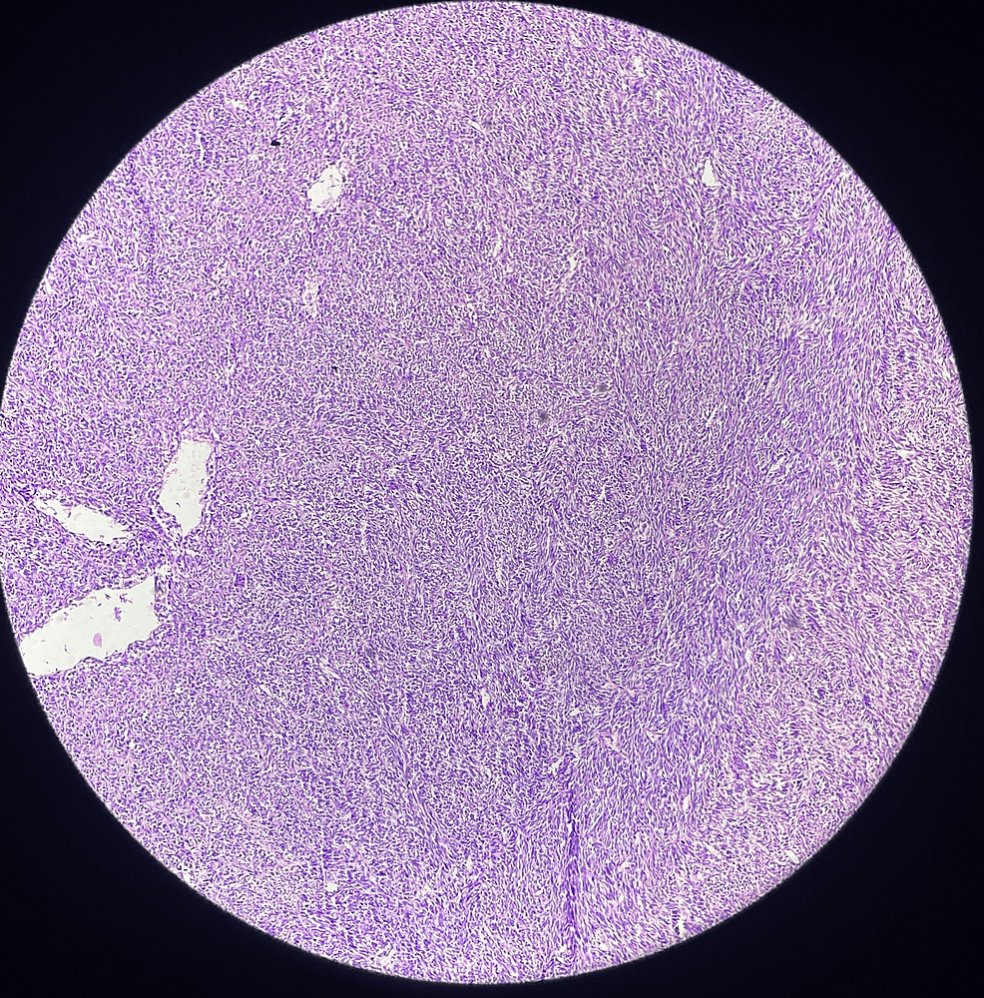
Hematoxylin and eosin slide image of the tumor (10x)

Ipsilateral superficial inguinal lymph nodes on the frozen section were negative for metastasis. The excised specimen along with the skin and superficial inguinal lymph nodes were sent for histopathology (Figure 6).

It was found that the tumor specimen which was 20 x 15 x 5 centimeters appeared soft and cystic in consistency, with reddish brown fluid oozing out along with grey, a white solid area of 10 x 8 x 3.5 centimeters with papillary excrescences. The mitotic rate was 1-3/10 HPF with the presence of necrosis, the tumor was histologically Grade I. Lympho-vascular space invasion and perineural invasion were absent. Margins of the tumor and superficial inguinal lymph nodes were negative for invasion by malignant cells except positive for dermis invasion. It was staged as pT4N0M0, stage IB. The primary course of treatment is still total surgical resection. On follow-up, the patient came with a healthy suture site. He received adjuvant radiotherapy to the tumor bed with adequate margins to a dose of 60 Gy in 30 fractions using parallel opposing portals with daily image guidance using cone beam CT. The testicular shielding was used during radiotherapy to reduce scattered dose to bilateral testes in this young gentleman to preserve fertility. He had tolerated treatment well with grade I radiotherapy-induced dermatitis. On the last follow-up after six months after his treatment completion, he was disease-free.

## Discussion

A form of angiosarcoma (hemangioendothelioma) with comparatively low biological aggressiveness has been identified as hemangiopericytoma (HPC), an unusual mesenchymal tumor. It can arise anywhere there are capillaries, leading to numerous particular issues with diagnosis and treatment [2]. Pericytes, contractile spindle cells that surround capillaries and post-capillary venules, are thought to be the source of this condition [4,5]. The immunohistochemical profile of hemangiopericytoma is ambiguous, and the diagnosis is frequently debatable. An issue is differentiating a tumor from synovial sarcoma, mesenchymal chondrosarcoma, fibrous histiocytoma, and solitary fibrous tumor [6]. Hemangiopericytoma is a rare adult-onset (fifth decade) tumor that is uncommon in kids. Less than 10% of all hemangiopericytomas (Enzinger and Smith 1976) 11 and roughly 3% of all soft tissue sarcomas in this age range are caused by pediatric instances [7]. Backwinkel compiled 224 examples from the literature that had received sufficient follow-up in 1969 [8]. This term was first used in 1942 by Stout and Murray to separate this perplexing tumor of blood vessel origin from the nearly related benign glomus tumor and other types of angiosarcomas. The hemangiopericytoma is made up of capillaries that are growing and spindle-shaped cells that resemble Zimmerman pericytes [2,9]. Stout believed that the main cell type in both tumors was pericytes. These cells, which are typically seen in the vicinity of capillaries, are thought to represent a particular kind of cell that resembles smooth muscle cells but lacks contractile fibers [3,9]. Hemangiopericytoma typically manifests as a painless mass, but symptoms could develop if the mass puts pressure on nearby viscera, and the likelihood of local recurrence is limited. Hemangiopericytoma has been linked to a number of paraneoplastic diseases, such as hypoglycemia and hypophosphataemicosteomalacia [10,11]. Hemangiopericytomas typically stain positively for markers such as CD34, CD99, vimentin, and smooth muscle actin, which helps distinguish them from other vascular tumors. Histologic confirmation is provided by the standard hematoxylin and eosin stain, with distinction aided by the silver reticulin stain [5,10,12]. According to Espat’s findings, patients who underwent total tumor excision had a 100% five-year survival rate [13]. However, other experts advise against procedures that could possibly result in loss of function or are limb-threatening given the favorable outcome in this condition. Vascular embolization of afferent arteries preoperatively may reduce the incidence of intra-operative bleeding. It is advised for all patients with incomplete resection and/or large, locally invasive tumors to get both chemotherapy and radiotherapy, as both treatments appear to be successful. In this case, the patient was treated by complete surgical extraction of the tumor with histopathological confirmation as hemangiopericytoma followed by adjuvant radiotherapy to avoid recurrence of the tumor.

## Conclusions

A slow-growing, cancerous tumor called a hemangiopericytoma has the potential to invade nearby tissue and spread through the circulation. The malignant and benign hemangiopericytomas cannot be reliably distinguished by a single histologic parameter. In some cases, a single surgical extraction might result in recurrence; hence, a wide excision, which might even involve amputation in some cases is preferable. Radiation therapy can be considered to have the potential to offer pain relief for non-resectable lesions due to its location, size, involvement with critical structures, or proximity to vital organs, furthermore, it can also be helpful in avoiding the recurrence of lesions after resection.
